# Photon-Counting Micro-CT for Bone Morphometry in Murine Models

**DOI:** 10.3390/tomography11110127

**Published:** 2025-11-13

**Authors:** Rohan Nadkarni, Zay Yar Han, Alex J. Allphin, Darin P. Clark, Alexandra Badea, Cristian T. Badea

**Affiliations:** Quantitative Imaging and Analysis Lab, Department of Radiology, Duke University Medical Center, Durham, NC 27710, USA; rohan.nadkarni@duke.edu (R.N.); zayyar.han@duke.edu (Z.Y.H.); alex.allphin@duke.edu (A.J.A.); darin.clark@duke.edu (D.P.C.); alexandra.badea@duke.edu (A.B.)

**Keywords:** photon-counting CT, mice, bone

## Abstract

Background/Objectives: This study evaluates photon-counting CT (PCCT) for the imaging of mouse femurs and investigates how APOE genotype, sex, and humanized nitric oxide synthase (HN) expression influence bone morphology during aging. Methods: A custom-built micro-CT system with a photon-counting detector (PCD) was used to acquire dual-energy scans of mouse femur samples. PCCT projections were corrected for tile gain differences, iteratively reconstructed with 20 µm isotropic resolution, and decomposed into calcium and water maps. PCD spatial resolution was benchmarked against an energy-integrating detector (EID) using line profiles through trabecular bone. The contrast-to-noise ratio quantified the effects of iterative reconstruction and material decomposition. Femur features such as mean cortical thickness, mean trabecular spacing (TbSp_mean), and trabecular bone volume fraction (BV/TV) were extracted from calcium maps using BoneJ. The statistical analysis used 57 aged mice representing the APOE22, APOE33, and APOE44 genotypes, including 27 expressing HN. We used generalized linear models (GLMs) to evaluate the main interaction effects of age, sex, genotype, and HN status on femur features and Mann–Whitney U tests for stratified analyses. Results: PCCT outperformed EID-CT in spatial resolution and enabled the effective separation of calcium and water. Female HN mice exhibited reduced BV/TV compared to both male HN and female non-HN mice. While genotype effects were modest, a genotype-by-sex stratified analysis found significant effects of HN status in female APOE22 and APOE44 mice only. Linear regression showed that age significantly decreased cortical thickness and increased TbSp_mean in male mice only. Conclusions: These results demonstrate PCCT’s utility for femur analysis and reveal strong effects of sex/HN interaction on trabecular bone health in mice.

## Simple Summary

Micro-CT imaging of bones from mice teaches us how genetics influence bone health. However, prior studies used micro-CT scanners with energy-integrating detectors and did not sufficiently explore the impact of sex and a humanized immune response (HN). This study used our photon-counting detector (PCD) micro-CT system to scan femurs from 57 mice with variations in apolipoprotein E (APOE) genotype, sex, and HN expression. The PCD reduced image blurring. The combination of female sex and HN expression was associated with sparser trabecular bone. Our work showed the promise of PCD imaging and highlighted complex risk factors for impaired bone health.

## 1. Introduction

Bone imaging is critical to understanding the interplay between skeletal integrity and systemic factors such as aging, metabolism, inflammation, and genetic predisposition. X-ray CT is typically used for these studies due to its inherent high contrast for bone imaging. While many previous studies have used conventional micro-CT systems with energy-integrating detectors (EIDs) to quantify bone architecture, this type of detector has limitations in spatial resolution, beam hardening, and compositional differentiation. Photon-counting computed tomography (PCCT), in contrast, represents a transformative advance. Conventional energy-integrating detectors (EIDs) typically use scintillators to convert X-ray photons into visible light and then into electrical signals, whereas photon-counting detectors (PCDs) directly convert X-ray photons into electrical pulses that are individually counted according to user-defined energy thresholds [1,2]. This results in improved spatial resolution, reduced electronic noise, and multi-energy imaging in a single acquisition [2].

PCCT’s potential for bone imaging has been demonstrated in both clinical and preclinical studies. In cadaveric human bones, PCCT has been shown to improve tissue contrast and reduce metal artifacts as compared to dual-energy EID CT [3,4]. Preclinical work using PCCT in rodent models has also shown accurate material decomposition and significant detection of disease-related changes, such as those induced by ovariectomy [5]. However, the application of PCCT to bone morphometry in genetically diverse murine models has not yet been fully explored.

Bone health is interconnected with the cardiovascular and neurological systems through shared pathways involving apolipoprotein E (APOE) and nitric oxide (NO) [6,7,8,9]. APOE, a lipid-transport protein with three isoforms (ε2, ε3, and ε4), influences bone formation via lipid metabolism [6]. Genetic variation or deficiency in APOE has been linked to altered bone remodeling [9,10]. While some studies report associations between ε4 and reduced bone mineral density [6,11,12], others find no clear link [13,14], suggesting context-dependent effects.

Mouse models are useful for studying how interactions between risk factors influence bone health because both their genetic background and lifestyle factors such as diet and exercise can be tightly controlled. While prior studies on bone health have used mice with variations in APOE genotype [15,16], incorporating additional variation in immune response via the presence or absence of a humanized NOS2 (HN) gene [17] is much less common. NOS2 regulates nitric oxide (NO) production during inflammation, affecting bone remodeling via osteoblast/osteoclast activity and oxidative stress [18].

In this study, we leverage a custom PCCT system to image femurs from 57 aged mice with defined homozygous APOE genotypes (APOE22, APOE33, and APOE44), including a subset expressing humanized NOS2. Using calcium maps derived from multi-energy acquisitions, we quantify the trabecular and cortical bone features and examine group-level trends linked to sex, immune status, APOE genotype, and age. We previously presented preliminary results from this study at the 2025 SPIE Medical Imaging conference [19]. The current manuscript demonstrates the full results following the completion of this study. These expanded results include a demonstration of the technical advantages of PCCT for bone imaging relative to EID CT and more in-depth stratified analyses that investigate the genotype/HN/sex triple-interaction effects as well as the effects of age on femur features. This work highlights PCCT’s advantages for musculoskeletal research and applies PCCT imaging of mouse femurs to the study of the complex interactions between risk factors for impaired bone health.

## 2. Materials and Methods

### 2.1. Mouse Cohort and Sample Preparation

**Figure A1:**
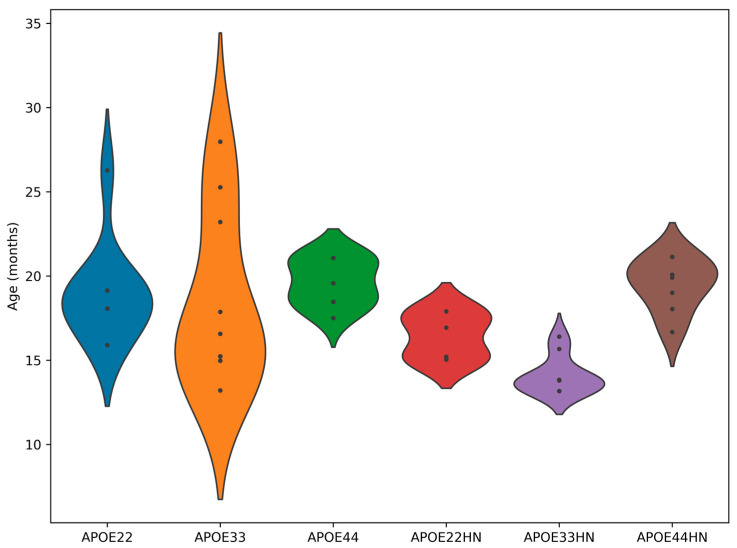
Violin plots showing distribution of age (in months) within each APOE × HN subgroup. The points inside each violin show the unique values of age within that subgroup.

This study included femurs from 57 aged C57BL/6J mice, which are the same mice previously analyzed in our preliminary version of this study [19]. These mice were genetically engineered to express one of three human APOE alleles (ε2, ε3, or ε4) in homozygous form, resulting in 3 distinct APOE genotypes (APOE22, APOE33, and APOE44). Of these, 27 mice (47%) also expressed a humanized version of the NOS2 gene (HN), providing an immune background more representative of human physiology [17,20]. The cohort included both sexes and spanned an age range of 13.2 to 28.0 months (mean ± SD: 17.8 ± 3.3 months). All animals were maintained on a standard chow diet (LabDiet 5001) and had an average body mass of 31.1 ± 4.1 g at the time of imaging. Table 1 summarizes the distribution of mice by genotype, sex, and HN status. Table 2 provides the corresponding age distributions (mean ± std dev), and Figure A1 shows violin plots with the complete range of age values within each APOE × HN subgroup.

This cohort of 57 mice gives us between 8 and 11 mice per subgroup when running statistical comparisons that group by combinations such as APOE genotype/HN status or APOE genotype/sex. Therefore, the cohort size is sufficient to achieve similar statistical power as previous work in the micro-CT imaging of bones from humanized APOE mice [15], which compared differences in femur features only by APOE genotype with 10 femurs per genotype.

While all APOE × HN subgroups included both sexes, complete age- and sex-matching was not possible due to breeding constraints. These constraints include (1) variations in litter size and breeding intervals between the different APOE genotypes that makes it difficult to achieve perfect age matching in a reasonable timeframe and (2) our use of each mouse in studies of multiple organs, which forces us to compromise between age, sex, and genotype balance in several studies when choosing the age at which to sacrifice our mice. Although Table 2 does show minor age differences, Figure A1 shows that there is a great degree of overlap in age range between the APOE × HN subgroups. We also quantified the age range overlap between groups using the Jaccard index, defined as the ratio of the intersection over the union (IoU), and included these results in Appendix A. Table A1 shows Jaccard indices between APOE genotypes, while Table A2 shows Jaccard indices for the comparison of HN and non-HN mice both within each genotype and across the entire cohort of mice without considering APOE genotype.

All animal procedures were approved by the Duke University Institutional Animal Care and Use Committee (IACUC, Protocol Registry Number: A173-20-08). Mice were euthanized by transcardiac perfusion under deep anesthesia induced by an intraperitoneal injection of ketamine/xylazine (100 mg/kg and 10 mg/kg, respectively), as previously described [21]. We ensured that this euthanasia was performed humanely with concern for the welfare of the mice. The left femur was excised, and soft tissues were carefully removed. Although there was variation in the size of the femur between individual mice, each femur was roughly 15 to 17 mm in length with a major axis diameter of about 2 mm at the mid-shaft. Femurs from three different mice were embedded in 1% agarose gel (w/v in PBS), with rubber bands serving as fiducial markers to help us differentiate between the femurs. Once the gel solidified, it was placed inside a 15 mL conical tube (Falcon^TM^ centrifuge tube (Thermo Fisher Scientific (Waltham, MA, USA)), 17 mm diameter, 120 mm length) that was filled with phosphate-buffered saline (PBS) to preserve hydration and reduce beam hardening during our CT acquisition. The preparation followed recommendations for ex vivo murine femur imaging [22]. Following the completion of the sample preparation, each vial containing femurs was scanned using PCCT.

### 2.2. Photon-Counting CT Scanning

**Figure A2:**
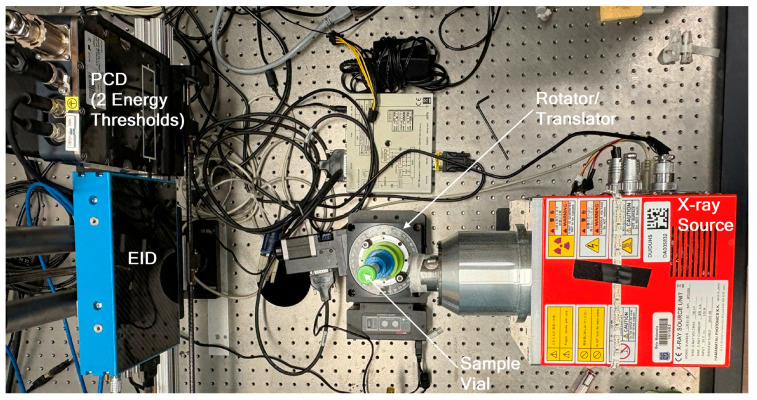
Labeled photo of the micro-CT system. The vial containing femurs (sample vial) is mounted to a motorized stage that is used for both rotation and vertical translation of the vial (rotator/translator). A linear actuator allows the user to position the detector of choice behind the sample for imaging [23].

All ex vivo imaging was performed using a custom-built micro-CT scanner equipped with two X-ray detectors: a photon-counting detector (PCD) and a conventional energy-integrating detector (EID) [23]. A labeled photo of our imaging setup is shown in Figure A2. The PCD (XC-Thor, Direct Conversion, Stockholm, Sweden) consists of 8 tiled CdTe-based detector chips with dimensions of 128 × 256 pixels per tile (1024 × 256 for the whole detector), a pixel size of 100 μm, and two programmable energy thresholds per acquisition. The EID module (Dexela 1512, PerkinElmer, Shelton, CT, USA) features a 75 μm pixel size and uses a CsI scintillator coupled with a CMOS sensor. Both the PCD and EID imaging chains of our micro-CT system have a magnification of about 5.3. The PCD was the primary detector used for femur imaging and material decomposition. The EID was included in this study solely for a comparison of spatial resolution, as detailed in our previous studies [20,23]. Both detectors were mounted orthogonally to the X-ray source and could be interchanged without altering the scanning geometry [23]. For all PCD scans, the X-ray source was operated at 60 kVp and 134 μA. Data were acquired using a step-and-shoot protocol covering 1400 angular views over 1070° of total rotation with 21 mm of vertical translation. At each projection angle, 100 low-noise exposures of 40 ms were summed. The first PCD energy threshold was set to 15 keV to reject electronic noise and low-energy scattered photons, while the second threshold was set to 34 keV (near the midpoint of the 60 kVp spectrum) to maintain sufficient photon counts and optimize sensitivity for calcium and water separation in subsequent material decomposition. With these settings, each vial scan required approximately 2 h and 6 min to complete.

### 2.3. Artifact Correction

The 8 detector tiles of the XC-Thor PCD have varying spectral responses. As a result, the projections have noticeable intensity differences between tiles even after log-normalization, and low-frequency concentric bands are present in the reconstructed image [24]. As we have discussed in our prior study on ex vivo brain imaging [20], we correct this using the multiplicative projection domain water gain correction proposed by Kim and Baek [25], with a PCCT scan of a PBS vial used in place of a water vial scan. After this correction of background nonuniformities, the PCD projections from a femur sample vial are ready for reconstruction.

### 2.4. Image Reconstruction

Since analytical reconstruction of the corrected PCCT projections of a femur sample vial with the weighted filtered backprojection (wFBP) algorithm [26] results in a noisy image, iterative reconstruction is necessary. Accordingly, we performed iterative reconstruction of our PCD projections with two energy thresholds using the Multi-Channel Reconstruction (MCR) Toolkit, version 1 [27]. Our reconstructed volumes have an isotropic voxel size of 20 μm and about 1000 axial slices with dimensions of 960 × 960 voxels, although there was some variation in the values of these dimensions to account for changes in the position of the vial and the femurs inside it. For iterative reconstruction, we used the split Bregman method with the add-residual-back strategy [28] and rank-sparse kernel regression regularization (RSKR) [27,29], solving the following optimization problem:

(1) $$X\;=\;\underset{X}{\mathrm{arg min}}\frac{1}{2}\;\sum_{e}{\left||\;RX(e)\;-\;Y(e)\;|\right|}_{2}^{2}\;+\;\lambda {\left||\;X\;|\right|}_{BTV}.$$

Thus, we solve iteratively for the vectorized, reconstructed data (the columns of *X*) for each energy simultaneously (indexed by *e*). The reconstruction for each energy minimizes the reprojection error (*R*, system projection matrix) relative to the log-transformed PCD projection data acquired at each energy (the columns of *Y*). To reduce noise in the reconstruction, the data fidelity term is minimized subject to the bilateral total variation (*BTV*) measured within and between energies via RSKR. The regularization parameter, *λ*, is implicitly determined by local noise level estimation during the reconstruction process [27]. Each set of 2 energy channel, tile artifact-corrected PCD projection data from a femur sample scan was reconstructed using this approach with 4 iterations.

### 2.5. Material Decomposition

We performed image-based material decomposition of the PCD iterative reconstructions using the method of Alvarez and Macovski [30]. Thus, we performed a post-reconstruction spectral decomposition with H_2_O and calcium (Ca) as the basis functions:

(2) $$X\left(e\right)=\;C_{H2O}M_{H2O}\left(e\right)+\;C_{Ca}M_{Ca}\left(e\right).$$

In this formulation, *M* is a matrix of material sensitivities (attenuation per unit concentration for each material) at each energy. We computed the values in *M* by scanning and reconstructing a phantom containing a water vial and a vial of 40 mg/mL Ca in water and fitting the slope of attenuation measurements taken in the vials. *C_H2O_* represents the density in g/mL for H_2_O, while *C_Ca_* is the Ca concentration in mg/mL. Finally, *X* is the attenuation coefficient of the voxel under consideration at energy *e*. Material decomposition was performed by matrix inversion, solving the following linear system at each voxel:

(3) $$C=XM^{-1}$$

Orthogonal subspace projection was used to prevent negative concentrations [29]. Post-decomposition, the material maps were assigned to colors and visualized in ImageJ (version 2.16.0).

### 2.6. Image Quality Assessment

To compare spatial resolution between the photon-counting and energy-integrating detectors, we acquired additional scans of a representative mouse femur. This sample was scanned using both the PCD and EID of our micro-CT system described in Section 2.2 with the same helical trajectory and matched acquisition settings: 60 kVp tube voltage, 134 μA current, and an acquisition time of 1.2 s per projection angle (average of 12 projections with an exposure time of 100 ms each for the EID, sum of 30 projections with an integration time of 40 ms each for the PCD), which is the maximum allowable for the EID without saturation. The PCD scan used energy thresholds of 15 and 34 keV. Both images were reconstructed at 20 μm isotropic voxel size using our iterative reconstruction pipeline to enable the direct comparison of PCD and EID data. We measured the intensity along the same line profile through trabecular bone in both the PCD and EID reconstructions. We then normalized both line profiles by their maximum value and plotted them together to provide a visual assessment of the spatial blurring of the trabecular bone.

Using one of our femur samples, we compared image contrast between the PCD wFBP reconstruction, PCD iterative reconstruction, and material decomposition of the PCD iterative reconstruction. We computed the contrast-to-noise ratio (CNR) for each displayed image (PCD 15 keV and PCD 34 keV from both wFBP and iterative reconstructions, water and calcium) using the following formula:

(4) $$CNR=\;\frac{\mu_{1}-\mu_{2}}{\sqrt{\frac{\sigma_{1}^{2}+\sigma_{2}^{2}}{2}}}$$

where *μ*_1_ and *σ*_1_ are the mean and standard deviation from a foreground region of interest (ROI) in trabecular bone and *μ*_2_ and *σ*_2_ are the mean and standard deviation from a background, non-bone ROI inside the femur.

### 2.7. Femur Feature Extraction

Following the iterative reconstruction and material decomposition of each scan, we extracted quantitative features from the calcium material map for each femur using ImageJ and its BoneJ plugin [31]. First, we created separate image volumes that isolate each individual femur in the sample vial by creating a substack of axial slices from a cropped rectangular region of interest in the calcium map. Using this smaller, femur-specific volume, we followed the instructions from use case 2 in the BoneJ manuscript [31] to align the femur to its principal axes and compute mean cortical thickness in two dimensions (MeanThick2D) and in three dimensions (MeanThick3D) from the central axial slice of the femur.

Next, we analyzed the trabecular bone in the metaphyseal region located slightly above the growth plate of the distal femur, as suggested in previous studies of mouse femurs [22,32]. Specifically, we selected a region that is 80 axial slices (1.6 mm) in height, with the bottom slice located 40 slices (0.8 mm) above the center of the growth plate. After extracting a metaphyseal substack from the femur-specific volume, we used the freehand selection tool in ImageJ to draw manual contours of the region inside the cortical bone on every 10th axial slice, and then we interpolated the ROIs. This results in an 80-slice volume of interest (VOI) with a shape that matches the region inside the cortical bone. Figure 2 in our prior conference manuscript [19] shows the location at which we compute cortical thickness, as well as the location of the metaphyseal region and the contoured trabecular VOI inside this region.

By using the “clear” and “clear outside” functions in ImageJ, we defined separate volumes for the trabecular bone inside the contoured VOI and the cortical bone outside the VOI. We performed Otsu’s thresholding on each of these volumes to generate a trabecular mask and a cortical mask, then added them together to create a combined trabecular and cortical mask. The trabecular mask was passed into BoneJ to compute the trabecular bone volume and surface area. Bone volume was divided by the total volume of the 80-slice contoured VOI to compute the bone volume fraction (BV/TV). Trabecular spacing (TbSp) and trabecular thickness (TbTh) maps were defined by passing the combined trabecular and cortical mask into the BoneJ thickness function and then clearing the region outside the contoured VOI in the resulting maps. For each of these maps, we then computed the mean across all voxels that do not have values of 0 or NaN to obtain the TbSp_mean and TbTh_mean. Finally, the mean calcium concentration (MeanCaConc) was computed by multiplying the calcium map of the distal metaphyseal VOI by the trabecular mask and taking the mean across all voxels that have a non-zero value.

### 2.8. Statistical Analysis of Femur Features

After extracting the features described above from each femur, we used statistical methods to understand how these features are influenced by age, sex, APOE genotype, and immune status (HN). Our statistical analysis code, which is included in the online repository for this manuscript (https://gitlab.oit.duke.edu/rohan.nadkarni/pcct-femur-analysis/ (accessed on 16 September 2025)), used the scipy and statsmodel packages in the Python programming language. The following subsections describe the details of our generalized linear models and stratified tests investigating sex/HN interactions, which were initially reported in our preliminary study [19]. In addition, we describe our stratified tests investigating (1) HN effects in genotype-by-sex subgroups and (2) age effects, which are new analyses that are unique to the current manuscript.

#### 2.8.1. Tests for Normality and Homogeneity of Variance

First, we checked whether each feature followed a normal (bell-shaped) distribution using the Shapiro–Wilk test [33]. In the Shapiro–Wilk test, the null hypothesis is a normal distribution, the alternative hypothesis is a skewed distribution, and the significance level we chose for rejection of the null hypothesis was 5%. Next, we used Levene’s test to check if each femur feature meets the null hypothesis of homogeneity of variances across the experimental subgroups [34], with a 5% significance level for the rejection of this hypothesis. Table A3 in Appendix A shows the *p*-values from the Shapiro–Wilk and Levene’s tests. MeanThick2D, MeanThick3D, and MeanCaConc all had a *p*-value of greater than 0.05 in both tests, so we accept the null hypotheses that the feature is normally distributed and has equal variances across subgroups. BV/TV, TbTh_mean, and surface area did not meet the assumption of normal distribution but did meet the assumption of equal variances, while TbSp_mean failed both assumptions.

#### 2.8.2. Multi-Factor Generalized Linear Models

Each feature was analyzed using a generalized linear model (GLM), which can easily be adapted for normally distributed or skewed response data by adjusting its two main parameters: (1) the expected probability distribution family of the response variable and (2) the link function used to transform the response variable. Our consistent use of GLMs for both normally distributed and skewed data ensured that the predictors and types of model parameters (the coefficient and *p*-value for each predictor) were identical for all femur features.

Features that were normally distributed and had equal variances across subgroups were analyzed using a GLM with the Gaussian distribution family. The formula for the GLM was as follows:

Feature ~ Age + Sex + Genotype + HN + Age:Sex + Age:Genotype + Age:HN + Sex:Genotype + Sex:HN + Genotype:HN.

In this formula, Feature refers to a femur feature such as MeanThick2D, Genotype refers to the classification of mice by APOE with HN status ignored (APOE22, APOE33, or APOE44), and HN refers to classification of mice by HN status with APOE genotype ignored (HN or non-HN). In our model, age (in months) was treated as a continuous variable rather than as grouped bins, preserving variance information across the full cohort. The remaining 3 independent variables are categorical. For our categorical variables, the reference (level 0) values in our model were male, APOE33, and non-HN. We included all two-way interactions but excluded higher-order interactions from the GLM formula to simplify interpretation.

For features that failed the Shapiro–Wilk test or both tests, we applied a GLM with the Gamma distribution and a log-link function, which is suitable for positively skewed data [35]. These GLMs used the same formula as above.

For all GLMs, *p*-values were corrected for multiple comparisons using the Benjamini–Hochberg (BH) false discovery rate (FDR) procedure at a 5% threshold [36]. *p*-values corresponding to a single predictor across all femur features were grouped together during FDR correction. We report coefficients and corrected *p*-values for significant predictors only.

#### 2.8.3. Stratified Subgroup Analyses

**Figure A3:**
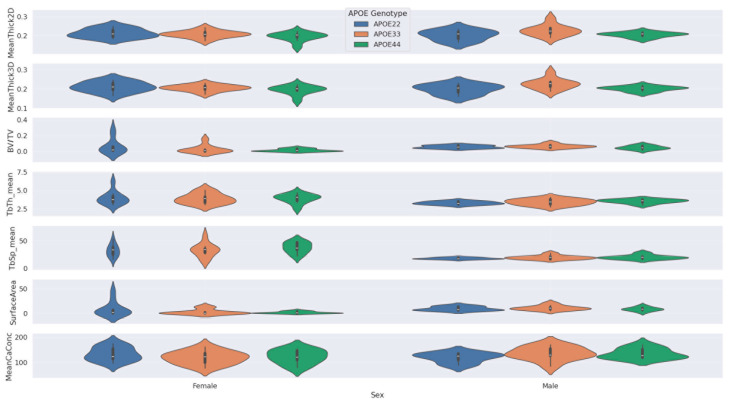
Violin plots corresponding to the Kruskal–Wallis comparisons by APOE genotype within the male and female subgroups. None of the plots include a bar with an asterisk because our Kruskal–Wallis tests did not find any femur features with significant difference by genotype within the male subgroup or the female subgroup.

**Figure A4:**
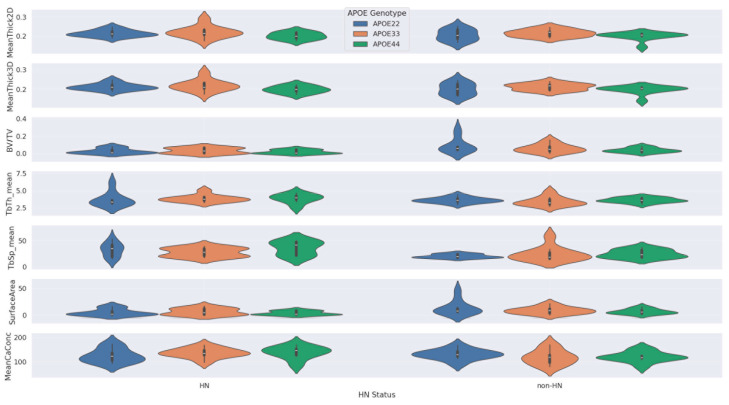
Violin plots corresponding to the Kruskal–Wallis comparisons by APOE genotype within the HN and non-HN subgroups. None of the plots include a bar with an asterisk because our Kruskal–Wallis tests did not find any femur features with significant difference by genotype within the HN subgroup or the non-HN subgroup.

In addition to running GLMs on the entire cohort of 57 mice, we ran several stratified subgroup analyses. First, we separated the data into male-only and female-only subgroups and assessed the effect of genotype on each femur feature within these subgroups (Figure A3). Then, we separated the data into HN and non-HN subgroups and assessed the effect of genotype on each femur feature within each of those groups (Figure A4). In these stratified analyses, we used the Kruskal–Wallis test [37] to check for differences by genotype, with the FDR correction of *p*-values using the BH method at a 5% significance threshold as described earlier. This test evaluates a null hypothesis of equal medians between three or more groups, making it suitable for femur features that are not necessarily normally distributed after stratification.

Our other stratified analyses investigated the effects of sex and HN on femur features. We sorted mice into HN and non-HN subgroups and determined if sex has an effect in each group. Conversely, we also sorted mice into male and female subgroups and determined if HN status has an effect in each group. For the femur features with significant difference by HN status in at least one sex-specific subgroup, we then sorted mice into genotype-by-sex subgroups (e.g., APOE22 Female) and assessed the effect of HN status in all six of these groups. Since HN and sex both have just 2 categories, we used the Mann–Whitney U test (Kruskal–Wallis test comparing only two groups) with BH FDR correction of *p*-values for each of these stratified tests.

Finally, we assessed the effect of age within stratified subgroups. Since age is a continuous variable in our analysis, this was achieved using linear regression. For each combination of femur feature and categorical variable (sex, APOE genotype, and HN), we generated a scatter plot with age on the x-axis and the femur feature (or its natural logarithm if it has a skewed distribution) on the y-axis, with separate color-coding and best-fit lines for each level (e.g., male and female) of the categorical variable. Unlike the multivariate GLMs discussed earlier, this stratified analysis fitted lines with the simple formula G(Feature) = m × Age + b, where G() refers to the log() operation for femur features with a skewed distribution and the identity operation for femur features with a normal distribution. *p*-values from these regressions were BH FDR-corrected across all features within each categorical level.

### 2.9. Qualitative Assessment of Trabecular Structure

To validate the accuracy of our trabecular metrics and provide a visualization of the differences between femur samples, we used ImageJ’s 3D Viewer plugin [38] to generate 3D renderings (volume rendering, resampling factor 2) of the combined trabecular and cortical bone mask from the distal metaphyseal region of several femur samples. Specifically, we compiled a figure to display two metaphyseal 3D renderings for each combination of sex, APOE genotype, and HN status. For each combination, we arranged the renderings so that the younger mouse is on the left and the older mouse is on the right. For each femur sample selected for rendering, we also display the corresponding bone volume fraction and age.

## 3. Results

### 3.1. Image Quality Assessment

**Figure 1:**
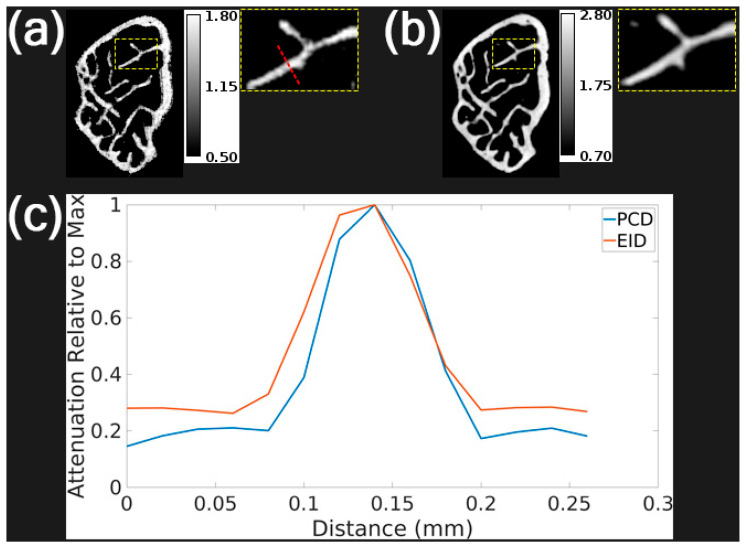
Spatial resolution assessment of PCD and EID scans on our ex vivo micro-CT system using a femur sample. (**a**) PCD iterative reconstruction of the femur sample at the first energy threshold. (**b**) EID iterative reconstruction of the same femur sample. (**c**) Plot of attenuation normalized by maximum value along the dashed red line profile in (**a**) in both PCD and EID reconstructions. The calibration bars in (**a**,**b**) show the display settings for these images in units of cm^−1^.

Figure 1 compares the spatial resolution of PCD and EID imaging using a mouse femur. In these femur images (Figure 1a,b), the PCD clearly delineates bone boundaries with reduced spatial blurring. The line profile analysis (Figure 1c) further confirms a sharper transition and narrower peak for the PCD image. To minimize noise in bone imaging, all PCCT femur scans in our aging study used a longer acquisition time per angle (4 s) than the data shown in Figure 1. This longer exposure remained within PCD dynamic range and did not introduce saturation.

**Figure 2:**
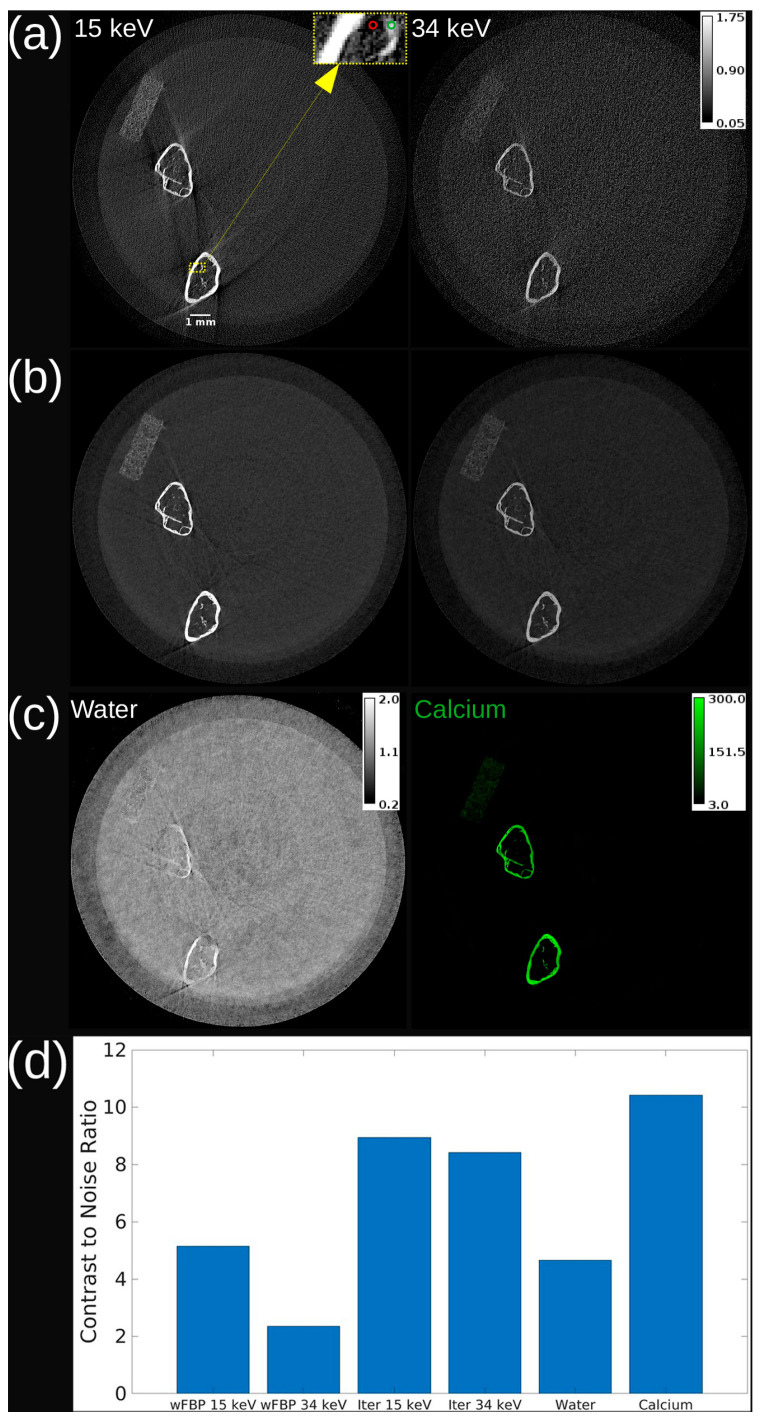
Axial slice from the reconstruction and material decomposition of a vial with mouse femurs. (**a**) wFBP reconstruction at both energy thresholds. (**b**) Iterative reconstruction at both energy thresholds. (**c**) Water and calcium maps from the material decomposition of iterative reconstruction. (**d**) Contrast-to-noise ratio for each image. The calibration bar in (**a**) shows the display setting for wFBP and iterative reconstructions in units of cm^−1^, while the calibration bars in (**c**) show the display settings of water in g/mL and calcium in mg/mL. The 15 keV threshold image in (**a**) includes a scale bar to indicate a length of 1 mm on the image. The area highlighted by a dashed yellow rectangle in (**a**) shows the foreground region in the trabecular bone (green circle) and the background region (red circle) used to calculate the CNR in all images. Note that although decomposition is very effective in separating the bone, some cross-contamination exists, and bone traces are apparent in the water image.

Figure 2 shows axial slices of a femur scan across three image types: PBS-corrected wFBP, iterative reconstruction, and material maps from decomposition. While the wFBP 34 keV image exhibits low CNR due to high noise, iterative reconstruction reduces the noise level and preserves bone structure at both energies. Material decomposition further enhances bone separation, with the calcium map achieving the highest CNR among all image types. Minor bone residuals in the water map are visible, likely due to partial volume effects or basis material limitations.

### 3.2. Statistical Analysis of Femur Features

#### 3.2.1. Multi-Factor Generalized Linear Models

Table 3 shows all significant predictors from our GLMs applied to the entire cohort of mice (*n* = 57).

Significant predictors were found only for trabecular bone volume fraction (BV/TV) and surface area. Notably, a change from the reference genotype APOE33 to APOE44 was associated with an increased BV/TV (*p* = 0.00976). The most pronounced effect was observed for the interaction between sex and HN status: female HN mice exhibited significantly lower BV/TV and surface area compared to both HN males and non-HN females (*p* < 1 × 10^−5^ for both features). Age also interacted with genotype, such that APOE44 mice showed a steeper decline in BV/TV with increasing age than APOE33 mice (*p* = 0.00518). While age was a significant predictor only in this interaction term, we note that the APOE44 and APOE44HN groups had slightly higher mean ages than the APOE33 and APOE33HN groups, which may have contributed to this result.

**Figure 3:**
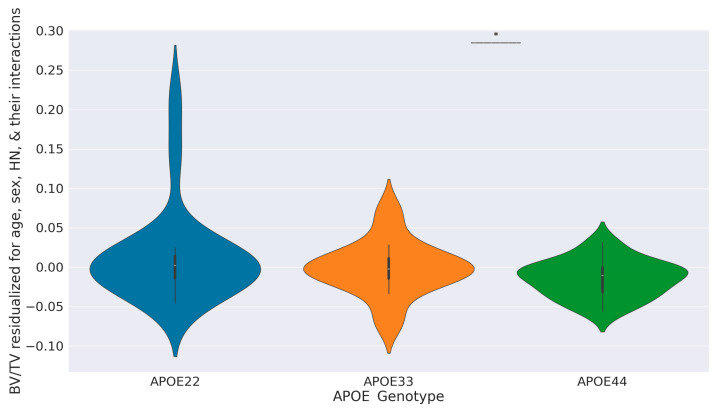
Violin plots showing differences in BV/TV by genotype. Rather than directly plotting BV/TV on the y-axis, we show the distribution of residuals from a confounding variables GLM with the formula of BV/TV ~ Age + Sex + HN + Age:Sex + Age:HN + Sex:HN for each APOE genotype. The bar with asterisk indicates a significant difference (*p* < 0.05) between APOE44 and the reference genotype level APOE33.

Although our GLM for BV/TV found a significant difference between APOE33 and APOE44, visual differences between genotypes were modest [19]. Therefore, to control for confounding variables, we computed BV/TV residuals from a GLM that excluded genotype effects. The violin plot of these residuals, displayed in Figure 3, shows more negative BV/TV residuals in APOE33 and APOE22 compared to APOE44, and a tighter distribution for APOE44.

These findings underscore the importance of sex, APOE genotype, and immune background in determining trabecular bone structure in aged mice.

#### 3.2.2. Stratified Subgroup Analyses

In our Kruskal–Wallis tests, we did not find a significant difference in femur features by APOE genotype within the male-only, female-only, non-HN-only, or HN-only subgroups. The violin plots associated with these results are shown in Figure A3 and Figure A4.

Our Mann–Whitney U tests comparing femur features by sex within the HN and non-HN subgroups and by HN status within the female and male subgroups returned several statistically significant differences. These significant results are reported in Table 4, and the median and interquartile range (IQR) of femur features in the corresponding sex/HN groupings are in Table 5 and Table 6.

The significant findings summarized in these three tables indicate that (1) female mice that express the HN gene have significantly less trabecular bone mass (i.e., smaller BV/TV and surface area and larger TbSp_mean) than male mice that express the HN gene, and (2) female mice that express the HN gene have significantly less trabecular bone mass than female mice that do not express the HN gene. However, our Mann–Whitney U tests do not show significant differences in femur features between the female non-HN and male non-HN groups or between the male HN and male non-HN groups.

Table 7 highlights the statistically significant findings from our Mann–Whitney U tests for differences in femur features by HN status within genotype-by-sex subgroups. Only three femur features were considered in this analysis—BV/TV, TbSp_mean, and surface area—since these were the only features that showed significant HN effects in our sex-stratified analysis (Table 4 and Table 6).

Significant differences by HN status were found for all three femur features in APOE22 females and for BV/TV and TbSp_mean in APOE44 females. In contrast, no significant differences by HN status were observed in APOE33 females or in any of the male subgroups.

**Figure 4:**
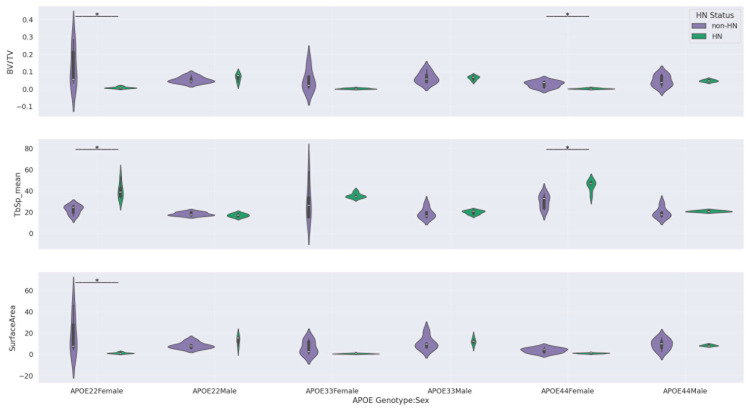
Violin plots corresponding to the Mann–Whitney U comparisons by HN status within genotype-by-sex subgroups. Bars with an asterisk indicate statistically significant differences (*p* < 0.05) by HN status after BH FDR correction. Only femur features that gave significant difference by HN status in at least one sex-specific subgroup in Table 4 and Table 6 were included in this figure.

These results indicate that the HN-associated bone loss in females is modulated by genotype, with APOE22 and APOE44 backgrounds showing the strongest effect. This three-way interaction (genotype/sex/HN) supports the notion that inflammatory signaling (modeled by HN expression) interacts with genetic susceptibility and sex to influence trabecular bone health in aging. However, we acknowledge that a broad distribution of ages within the group of APOE33 mice in our study (standard deviation of 5.12 months) may have contributed to the lack of significant HN effects in the APOE33 female group. The violin plots corresponding to the comparisons in Table 7 are shown in Figure 4, providing a visual summary of how HN effects differ by genotype and sex.

**Figure 5:**
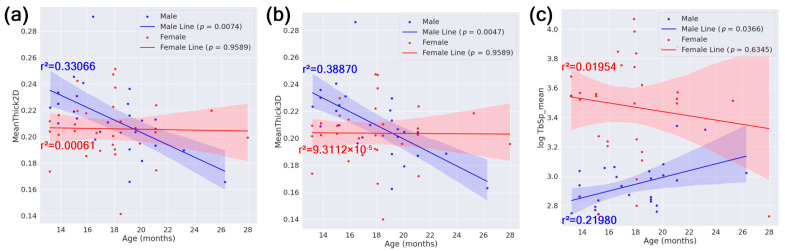
Scatter plots of femur feature vs. age. Linear regression plots with age as the independent variable, separate linear fits by sex, and (**a**) MeanThick2D, (**b**) MeanThick3D, and (**c**) log(TbSp_mean) as the dependent variable. Each plot includes best-fit lines as well as their r^2^ values, 95% confidence intervals, and *p*-values (in legend). For brevity, only plots with a statistically significant regression line (*p* < 0.05) in at least one subgroup are shown.

In our linear regression with age on the x-axis and femur feature on the y-axis within subgroups defined by sex, HN status, or APOE genotype, only the male subgroup showed statistically significant regression lines after BH FDR correction. Specifically, male mice showed a significant decrease in cortical thickness (both 2D and 3D measures) and a significant increase in trabecular spacing (log-transformed TbSp_mean) with increasing age. Scatter plots illustrating these relationships, including regression lines, r^2^ values, confidence intervals, and corrected *p*-values, are shown in Figure 5.

No other subgroups—females, HN mice, non-HN mice, or any APOE genotype groups—exhibited significant age-dependent changes in the examined femur features after multiple-testing correction. Furthermore, the interaction term Age:Sex was not significant in the full-cohort GLMs, suggesting that the age-related differences observed in males did not extend to females in this cohort.

These findings support a sex-specific aging trajectory, where male mice exhibit more pronounced bone deterioration with age, while female mice may be more affected by inflammatory and genetic interactions (e.g., HN and APOE effects).

### 3.3. Qualitative Assessment of Trabecular Structure

**Figure 6:**
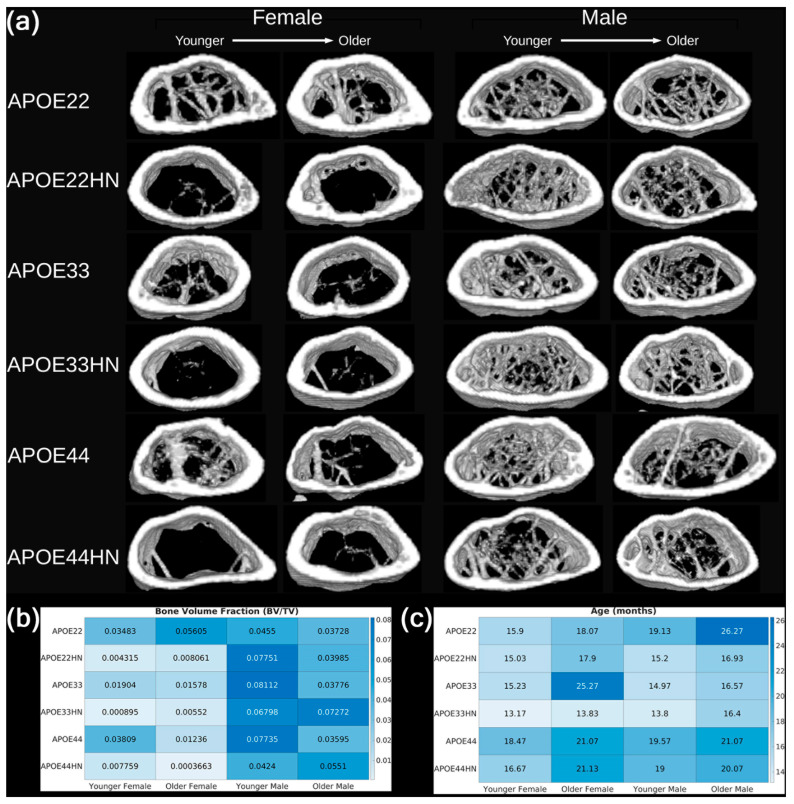
Qualitative assessment of trabecular bone in the metaphyseal region. (**a**) 3D renderings of combined trabecular and cortical masks from metaphyseal volumes of interest, with example bones from two mice shown for each combination of genotype, HN status, and sex. Heatmaps indicating (**b**) bone volume fraction and (**c**) age (in months) for each individual mouse whose bone is displayed in the 3D rendering plot in (**a**).

In Figure 6a, we show 3D renderings of the distal femoral metaphyseal region from 24 representative mice spanning all combinations of APOE genotype, sex, and HN status. These visualizations provide a qualitative perspective on trabecular bone morphology and help contextualize the quantitative metrics. Accompanying heatmaps display the corresponding bone volume fraction (BV/TV, Figure 6b) and age (Figure 6c) for each sample.

The 3D renderings confirm key trends observed in the statistical analysis. Female HN mice consistently exhibit sparser and more fragmented trabecular structures than either male HN mice or female non-HN mice. In contrast, male mice—both with and without HN expression—display more robust and interconnected trabecular networks. These observations are visually striking and align closely with the results from GLMs and subgroup analyses. Some differences between males and females in the non-HN group were also apparent in the renderings. However, these did not reach statistical significance, possibly due to the thicker trabeculae observed in female non-HN mice, which may partially offset the reduced number of trabecular elements. Both qualitative and quantitative inspection of Figure 6a reveal that in this group of aged mice, the effects of age on trabecular bone within each genotype/HN status/sex subgroup are negligible compared to the effects of sex difference, especially among HN mice.

Overall, the qualitative visualizations in Figure 6 reinforce the conclusion that sex and HN status jointly influence trabecular architecture, particularly in female mice.

## 4. Discussion

Our image quality assessments demonstrated the efficacy of the photon-counting CT-based femur image acquisition and processing procedure. Figure 1 displays equivalent-dose PCD and EID reconstructions from scans on our ex vivo micro-CT system that proved that the PCD has less spatial blurring of trabecular bone in a femur sample despite having a slightly larger pixel size than the EID (100 μm vs. 75 μm). We demonstrated in Figure 2 that material decomposition of the multi-energy PCD iterative reconstruction produces a calcium map that has higher CNR between the trabeculae and background compared to the PCD CT images. These results show that our combination of multi-energy photon-counting CT imaging and iterative reconstruction with joint regularization of energy channels works well. Material decomposition further enhanced image interpretability by isolating calcium content, which is directly correlated with bone density.

Using this pipeline, we quantified femur features (e.g., BV/TV, TbSp_mean, and MeanThick2D/3D) across 57 mice with variations in APOE genotype, sex, HN status, and age. While complete cohort matching of age and sex across all subgroups was not possible, our statistical modeling did incorporate age, sex, and their interactions as predictors of femur features. Through this modeling approach, we ensured that genotype and HN effects on femur features were evaluated while also accounting for how age may moderate these effects. Our GLMs identified significant sex/HN interaction effects on BV/TV and surface area and found APOE44 to be associated with increased BV/TV relative to APOE33 (Figure 3). The result for APOE44 mice is contrary to initial expectations that the ε4 allele—linked to neurodegeneration—would have negative effects on bone. While this finding diverges from younger cohorts in prior literature [15], it underscores the importance of studying aging-specific effects and considering immune context (HN status).

Stratified subgroup analyses confirmed pronounced sex-specific HN effects on trabecular bone mass. Specifically, female HN mice had significantly reduced BV/TV, increased trabecular spacing, and smaller trabecular surface areas compared to both male HN mice and female non-HN mice (Table 4, Table 5 and Table 6). This suggests that the humanized immune background in HN mice modulates sex differences in bone remodeling—likely via inflammatory or hormonal pathways [39]. Notably, these effects were not statistically significant in APOE33 females (Table 7, Figure 4), indicating a genotype-specific modulation of HN influence. While we acknowledged the younger age range of APOE33HN mice when discussing these results in APOE genotype-by-female subgroups, the results in Table A2 (limited to moderate age overlap between APOE33 HN and APOE33 non-HN) and Figure 5 (significant age effects on femur features in males but not females) suggest that our findings in APOE33 females are more likely to reflect a real impact of the APOE33 genotype rather than an unintended consequence of age differences.

As mentioned above, aging was associated with cortical thinning and increased trabecular spacing in males (Figure 5), but not females. This may reflect the earlier onset of bone loss in female mice, potentially masking progressive changes over time. Age–sex interaction terms were not significant in whole-cohort GLMs, possibly due to strong interactions with HN in female mice.

3D renderings of the trabecular architecture (Figure 6) visually reinforced quantitative findings, particularly the stark contrast between female HN and male HN bones. These renderings also suggest that statistical non-significance in some subgroups (e.g., female non-HN vs. male non-HN) may arise from a variation in trabecular thickness rather than the number of trabeculae.

From a biological perspective, our results emphasize that APOE genotype alone has limited predictive power for bone health, aligning with some human studies [14]. Instead, the interaction between genotype, sex, and immune status (HN) was critical in our study. These findings support the use of HN mice in preclinical studies of bone disease, especially for capturing sex-specific vulnerabilities.

Our study complements prior work using PCCT to evaluate cardiac function in APOE/HN mice [40]. Together, the cardiac and bone findings point toward systemic effects of APOE and immune background across multiple organ systems. Both studies implicate shared mechanisms, e.g., inflammation, oxidative stress, and lipid metabolism, driving organ remodeling with age. This supports the broader utility of PCCT as a multi-organ phenotyping platform in aging research.

One shortcoming that we acknowledge is that reconstructions from our custom-built micro-CT system (magnification ~5.3) have a larger voxel size (20 μm) than reconstructions from high-quality commercial EID-based micro-CT scanners (~5 μm). This is because compared to our system, these commercial scanners can typically achieve smaller focal spot sizes (reducing penumbra blurring), higher magnifications, and in some cases, smaller detector pixel sizes. On the other hand, the magnification of our system is constrained by the limited field-of-view of our PCD combined with our desire to perform high-throughput imaging by scanning multiple femurs (i.e., three) in each vial. Nevertheless, this study demonstrates that the properties of the PCD, such as reduced spatial blurring due to direct X-ray photon detection and simultaneous multi-energy imaging, are useful for bone imaging. Future work that incorporates the PCD into commercial micro-CT scanners that can achieve voxel sizes of less than 10 μm will be critical to ensure that the benefits of photon-counting CT for small animal bone imaging are fully realized.

Another potential shortcoming is the size (*n* = 57) and age/sex distribution of our study cohort. Although this sample size is sufficiently large for our whole-cohort GLMs and all stratified tests exploring double interactions (e.g., APOE genotype/sex, APOE genotype/HN, sex/HN, Table 4, Table 5 and Table 6, and Figure A3 and Figure A4), our triple-interaction analysis investigating HN effects in genotype-by-sex subgroups (Table 7 and Figure 4) may require validation in a larger mouse cohort. Future work in a larger study cohort with better matching of age distribution across all genotype/HN groups and a better balance of sexes in the APOE22HN and APOE44HN groups may also further improve the reliability of statistical analyses investigating effects of two or more interactions.

## 5. Conclusions

We present a validated PCD micro-CT pipeline for ex vivo bone imaging in aged mice. Our image quality assessments confirmed that the PCD provides higher spatial resolution than matched-dose EID images and that the material decomposition of PCD images improves the contrast-to-noise ratio. We used our PCCT pipeline to show that trabecular bone metrics are significantly impacted by interactions between sex and immune (HN) background, with the modulation of this effect by APOE genotype. These findings extend the utility of PCCT beyond single-organ applications and highlight the complex, multi-factorial influences on bone health in aging. The methodological and biological insights gained here lay the groundwork for future multi-organ studies using PCCT in preclinical models of aging and disease.

## Figures and Tables

**Table 1:** Distribution of mice in femur study by genotype/HN status and sex.

Genotype	Female	Male	Total
APOE22	5	5	10
APOE33	5	5	10
APOE44	5	5	10
APOE22HN	6	3	9
APOE33HN	5	5	10
APOE44HN	5	3	8
Total	31	26	57

**Table 2:** Age distribution for the entire mouse population and for subgroups by genotype/HN status and sex.

Group (# of Mice)	Age in Months (Mean ± Std Dev)
APOE22 (10)	18.88 ± 2.88
APOE33 (10)	18.45 ± 5.12
APOE44 (10)	19.63 ± 1.37
APOE22HN (9)	16.45 ± 1.32
APOE33HN (10)	14.06 ± 1.09
APOE44HN (8)	19.5 ± 1.54
Female (31)	17.74 ± 3.38
Male (26)	17.86 ± 3.19
All Mice (57)	17.79 ± 3.27

**Table 3:** Summary of significant predictors from GLMs.

Femur Feature	Predictor	Coefficient	BH FDR-Corrected *p*-Value	Interpretation
BV/TV	Genotype[T.APOE44]	9.08937	0.00976	APOE44 has a significant positive effect on log(BV/TV) relative to APOE33.
BV/TV	Sex[T.Female]:HN[T.HN]	−2.85595	<1 × 10^−5^	The combination of female sex and HN expression has a significant negative effect on log(BV/TV).
BV/TV	Age:Genotype[T.APOE44]	−0.484397	0.00518	The change in log(BV/TV) per 1-month increase in age is significantly more negative (i.e., more age-dependent decline) in the APOE44 group than in the APOE33 group.
Surface Area	Sex[T.Female]:HN[T.HN]	−2.19199	<1 × 10^−5^	The combination of female sex and HN expression has a significant negative effect on log(Surface Area).

**Table 4:** Summary of significant predictors from stratified subgroup analyses investigating sex and HN interactions. All *p*-values were computed using Mann–Whitney U tests followed by BH FDR correction.

Femur Feature	Predictor	Subgroup	BH FDR-Corrected *p*-Value	Interpretation
MeanThick2D	Sex	HN	0.03339	Significant difference in MeanThick2D between female HN mice and male HN mice.
BV/TV	Sex	HN	3.6770 × 10^−5^	Significant difference in BV/TV between female HN mice and male HN mice.
TbSp_mean	Sex	HN	3.6770 × 10^−5^	Significant difference in TbSp_mean between female HN mice and male HN mice.
Surface Area	Sex	HN	3.6770 × 10^−5^	Significant difference in surface area between female HN mice and male HN mice.
BV/TV	HN	Female	0.00050	Significant difference in BV/TV between female HN mice and female non-HN mice.
TbSp_mean	HN	Female	0.00060	Significant difference in TbSp_mean between female HN mice and female non-HN mice.
Surface Area	HN	Female	0.00060	Significant difference in surface area between female HN mice and female non-HN mice.

**Table 5:** Distributions of femur feature values in groupings corresponding to the Mann–Whitney U comparisons by sex within the HN and non-HN subgroups. For each comparison that returned a statistically significant difference (*p* < 0.05) in a femur feature by sex after BH FDR correction, we report the median and interquartile range (IQR) of that feature in each group. No significant differences in femur features by sex were found within the non-HN subgroup.

Femur Feature	HN Female Median and IQR	HN Male Median and IQR
MeanThick2D	0.20342 (0.01921)	0.22487 (0.02095)
BV/TV	0.00417 (0.00649)	0.06021 (0.02683)
TbSp_mean	37.559 (11.774)	20.231 (2.8949)
Surface Area	0.81193 (0.52843)	11.207 (6.019)

**Table 6:** Distributions of femur feature values in groupings corresponding to the Mann–Whitney U comparisons by HN status within the female and male subgroups. For each comparison that returned a statistically significant difference (*p* < 0.05) in a femur feature by HN status after BH FDR correction, we report the median and interquartile range (IQR) of that feature in each group. No significant differences in femur features by HN status were found within the male subgroup.

Femur Feature	Female HN Median and IQR	Female Non-HN Median and IQR
BV/TV	0.20342 (0.01921)	0.20614 (0.02302)
TbSp_mean	37.559 (11.774)	25.452 (12.233)
Surface Area	0.81193 (0.52843)	4.7195 (7.3122)

**Table 7:** *p*-values after BH FDR correction from the Mann–Whitney U comparisons by HN status within genotype-by-sex subgroups. *p*-values below the 5% significance level are indicated in bold text.

	APOE22 Female	APOE22 Male	APOE33 Female	APOE33 Male	APOE44 Female	APOE44 Male
BV/TV	**0.00433**	0.78571	0.09524	0.84127	**0.02381**	0.78571
TbSp_mean	**0.00433**	0.58929	0.15079	0.46429	**0.02381**	0.75000
Surface Area	**0.00433**	0.58929	0.15079	0.46429	0.05556	0.78571

## Data Availability

Data from this study is available on the following public Gitlab repository: https://gitlab.oit.duke.edu/rohan.nadkarni/pcct-femur-analysis (accessed on 16 September 2025).

## Appendix A

**Table A1:** Age overlap between APOE genotype pairs quantified using Jaccard index.

	APOE2	APOE3	APOE4
APOE2	1	0.76	0.40
APOE3	0.76	1	0.30
APOE4	0.40	0.30	1

**Table A2:** Age overlap between HN and non-HN mice within each APOE genotype and across the entire cohort, quantified using the Jaccard index.

APOE Genotype	Jaccard Index (HN vs. Non-HN)
APOE2	0.18
APOE3	0.22
APOE4	0.80
All	0.54

**Table A3:** Results of the Shapiro–Wilk and Levene’s tests on each femur feature.

Femur Feature	Shapiro–Wilk *p*-Value	Levene *p*-Value
MeanThick 2D	0.5626	0.8362
MeanThick 3D	0.5977	0.7124
BV/TV	<10^−4^	0.0556
TbTh_mean	0.0025	0.6208
TbSp_mean	0.0020	0.0360
Surface Area	<10^−4^	0.0908
Mean Ca Conc	0.1799	0.9911
